# Functional study of *CHS* gene family members in citrus revealed a novel *CHS* gene affecting the production of flavonoids

**DOI:** 10.1186/s12870-018-1418-y

**Published:** 2018-09-12

**Authors:** Zhibin Wang, Qibin Yu, Wanxia Shen, Choaa A. El Mohtar, Xiaochun Zhao, Fredrick G. Gmitter

**Affiliations:** 1 Citrus Research Institute, Southwest University, Xiema, Beibei, Chongqing, 400715 China; 2 Citrus Research and Education Center, University of Florida, 700 Experiment Station Rd, Lake Alfred, Florida, 33850 USA

**Keywords:** Chalcone synthase, Flavonoid, Gene expression, Gene silencing

## Abstract

**Background:**

Citrus flavonoids are considered as the important secondary metabolites because of their biological and pharmacological activities. Chalcone synthase (CHS) is a key enzyme that catalyses the first committed step in the flavonoid biosynthetic pathway. *CHS* genes have been isolated and characterized in many plants. Previous studies indicated that *CHS* is a gene superfamily. In citrus, the number of *CHS* members and their contribution to the production of flavonoids remains a mystery. In our previous study, the copies of *CitCHS*2 gene were found in different citrus species and the sequences are highly conserved, but the flavonoid content varied significantly among those species.

**Results:**

From seventy-seven *CHS* and *CHS*-like gene sequences, ten *CHS* members were selected as candidates according to the features of their sequences. Among these candidates, expression was detected from only three genes. A predicted *CHS* sequence was identified as a novel *CHS* gene. The structure analysis showed that the gene structure of this novel *CHS* is very similar to other *CHS* genes. All three *CHS* genes were highly conserved and had a basic structure that included one intron and two exons, although they had different expression patterns in different tissues and developmental stages. These genes also presented different sensitivities to methyl jasmonate (MeJA) treatment. In transgenic plants, the expression of *CHS* genes was significantly correlated with the production of flavonoids. The three *CHS* genes contributed differently to the production of flavonoids.

**Conclusion:**

Our study indicated that *CitCHS* is a gene superfamily including at least three functional members. The expression levels of the *CHS* genes are highly correlated to the biosynthesis of flavonoids. The CHS enzyme is dynamically produced from several *CHS* genes, and the production of total flavonoids is regulated by the overall expression of *CHS* family genes.

**Electronic supplementary material:**

The online version of this article (10.1186/s12870-018-1418-y) contains supplementary material, which is available to authorized users.

## Background

Flavonoids consist of over 7000 compounds and represent a large class of plant secondary metabolites [1–3]. In addition to being the primary compounds that determine the colour of flowers, fruits and leaves, flavonoids play important roles in protecting plants against damage from pathogens, pests and herbivores [4, 5], conferring resistance to abiotic stresses [6], and transporting plant hormones in diverse signalling pathways [7]. Flavonoids also have multiple benefits for human health [8], such as the prevention of cardiovascular and carcinogenic risks, promotion of antioxidant and anti-inflammatory activity, and protection against coronary heart disease and certain cancers [9–12].

Flavonoids are produced by all citrus species, such as mandarins, sweet or sour oranges, pummelos, grapefruits, limes and lemons [13]. Thus far, more than 60 flavonoid compounds have been identified in citrus. Those flavonoids can be classified into four major types of substances named flavones, flavonols, flavanones, and flavanonols according to their basic structures [14, 15]. Compared with other plant flavonoids, certain citrus flavonoids possess much stronger antioxidant activity due to their unique chemical structures [16–18].

The biosynthesis of flavonoids in plants has been well characterized [19, 20]. Although chalcone synthase (CHS) was identified as the first enzyme involved in the flavonoid biosynthesis pathway in 1972 [21], CHS was not reported in citrus until 1989 [22]. CHS controls the first committed step of flavonoid biosynthesis and catalyses three molecular malonyl CoA and one molecular 4-coumaroyl CoA into naringenin chalcone, which is then rapidly converted into naringenin (flavanone) by chalcone isomerase (CHI) and further synthesized into various flavonoids by the downstream enzymes involved in this pathway [23, 24]. Therefore, understanding the function of the *CHS* gene and its regulatory mechanism is vital to exploring the genetic control of this metabolite pathway.

In many dicots, CHS is encoded by a multigene family [25–27]. Usually, the chalcone synthase gene forms a family of three to twelve members in most of dicots, such as apple (3 members) [28], mulberry (5 members) [29], *Populus* (6 members) [30], *Glycine max* (8~ 9 members) [31, 32], *Viola cornuta* (10 members) [33], and petunia (12 members) [34]. In turnip, six *CHS* genes were cloned and identified, although only three were functional. The other three *CHS* genes were confirmed to be redundant genes [27]. In Valencia orange, two *CHS* (*CitCHS1* and *CitCHS2*) genes were identified by Southern blotting. The expression of the two *CHS* genes in relation to the biosynthesis of flavonoids was very different in citrus cell cultures. *CitCHS2* was found to strongly regulate the accumulation of flavonoids, but *CitCHS1* did not [35]. In our early study, the CDS (Coding Sequence) fragments of the *CitCHS2* gene cloned from ten different citrus species demonstrated high identity [36]. The analysis of flavonoid contents revealed significant differences among different species. However, a strong correlation between the expression of the *CitCHS2* gene and the accumulation of flavonoids is only present in a few species.

In the current study, seventy-seven *CHS* or *CHS-*like genes were studied to explore the structure and expression profile of the *CHS* gene family in citrus. The function of the *CHS* genes was verified by both overexpression and gene silencing via transgenic experiments. The *CHS* gene family and its activity in regulating the biosynthesis of flavonoids in citrus is discussed.

## Results

### Phylogenetic analysis of the *CHS* family genes

**Fig. 1:**
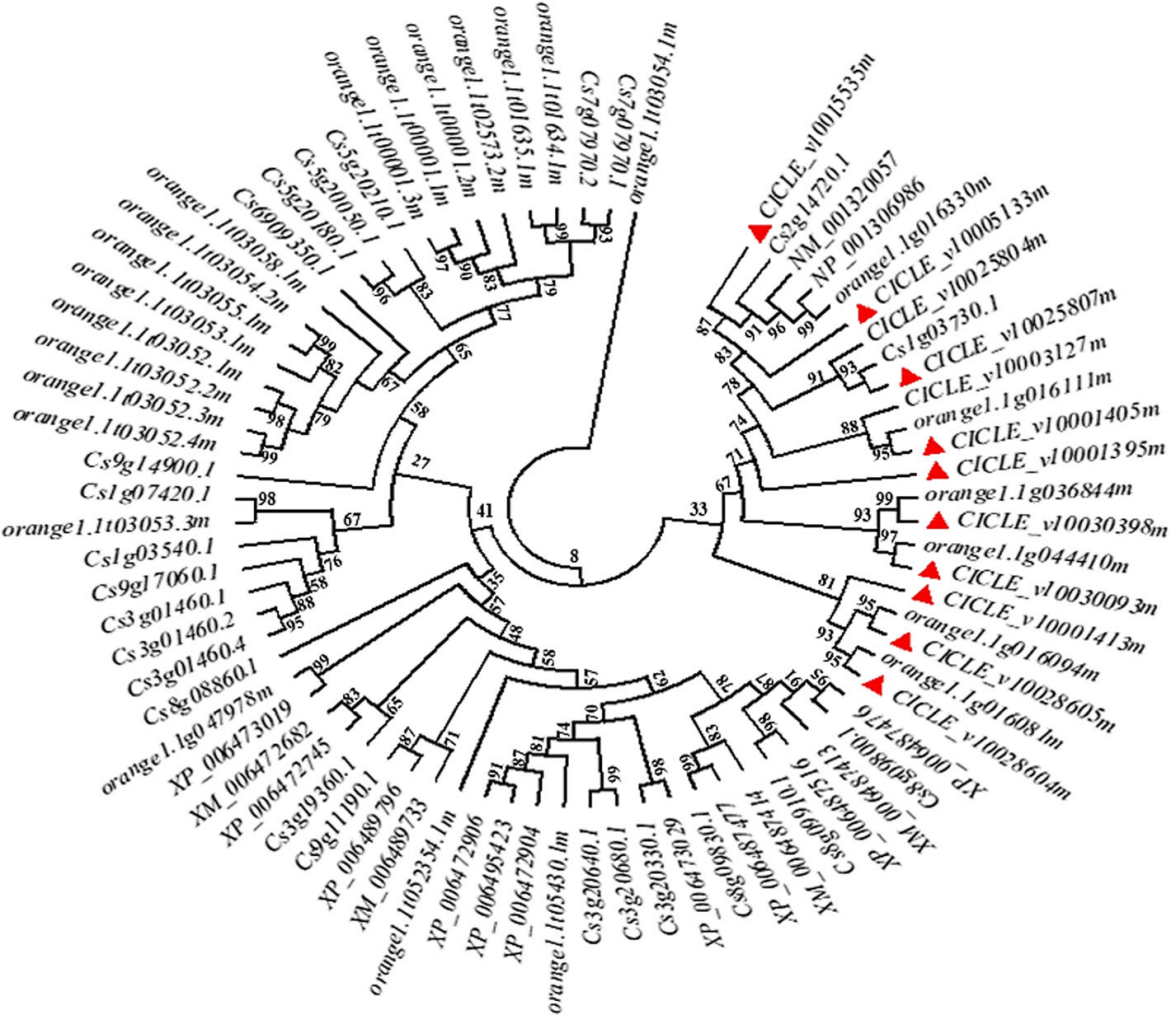
Phylogenetic tree based on the amino acid sequences of 77 chalcone synthase proteins. Among these sequences, 9 (XM_006487413.1, XM_006487414.2, XM_006472682.2, XM_006489733.1, NM_001320057.1, NM_001320057.1, XM_006489733.1, XM_006472682.2) were derived from NCBI, 16 (CICLE_v10001405 m, CICLE_v10001413 m, CICLE_v10003127 m, CICLE_v10005133 m, CICLE_v10015535 m, CICLE_v10028604 m, CICLE_v10028605 m, CICLE_v10030093 m, CICLE_v10030398 m, orange1.1 g016081 m, orange1.1 g016094 m, orange1.1 g016111 m, orange1.1 g016330 m, orange1.1 g036844 m, orange1.1 g044410 m, orange1.1 g047978 m) were derived from Phytozome and the remaining 52 were derived from the Orange Genome Annotation Project. The red triangle represents the selected candidate sequences used for the expression analysis. The alignments were saved and executed using MEGA version 7.1 to generate a neighbour-joining tree with a bootstrapping (1000 replicates) analysis, and gaps were addressed via pairwise deletion

The *CHS* gene has been reported to be a member of the PKS (Polyketide synthase) superfamily in plants [37]. A phylogenetic tree of the *CHS* family genes was constructed using the ClustalW method based on the substitution of amino acid residues of the *CHS* and *CHS*-like genes derived from citrus genome sequence databases (NCBI (https://www.ncbi.nlm.nih.gov/gene), Phytozome (https://phytozome.jgi.doe.gov/pz/portal.html) and Orange Genome Annotation Project (http://citrus.hzau.edu.cn/cgi-bin/orange/search)). The seventy-seven *CHS* and *CHS*-like genes were clustered into mainly three groups (Fig. 1). A more divergent structure of subgroups was found in group I. Group III was noticeably distant from group I and II. The genes in group I and II shared a higher similarity than they do with those in group III. The annotation indicated that most genes from group I and II are the non-functional NADPH-dependent codeinone reductase 2-like gene or type III polyketide synthase related genes. A high identity (75.0% to 88.2%, 1: Table S1) among *CitCHS1*, *CitCHS2* and other 20 genes in group III was observed, which suggested that the citrus *CHS* family may include many members.

### Expression profiles of *CHS* genes with or without MeJA treatment

To identify the functional members of the *CHS* family, ten candidate genes from each subgroups of group III of the phylogenetic tree were selected according to their similarity and structures and used for the gene expression analysis via qPCR with gene-specific primers (Additional file 1: Table S2). The transcripts were only detected from three genes with or without methyl jasmonate (MeJA) treatment. *CitCHS1* (CICLE_v10005133m) and *CitCHS2* (CICLE_v10015535m) were two of the three genes mentioned above. The third one, CICLE_v10001405m, has not yet been reported in any publication; it is distinct from the other two *CHS* genes and located in a different subgroup of the phylogenetic tree. This gene was named *CitCHS3* in the present study.

**Fig. 2:**
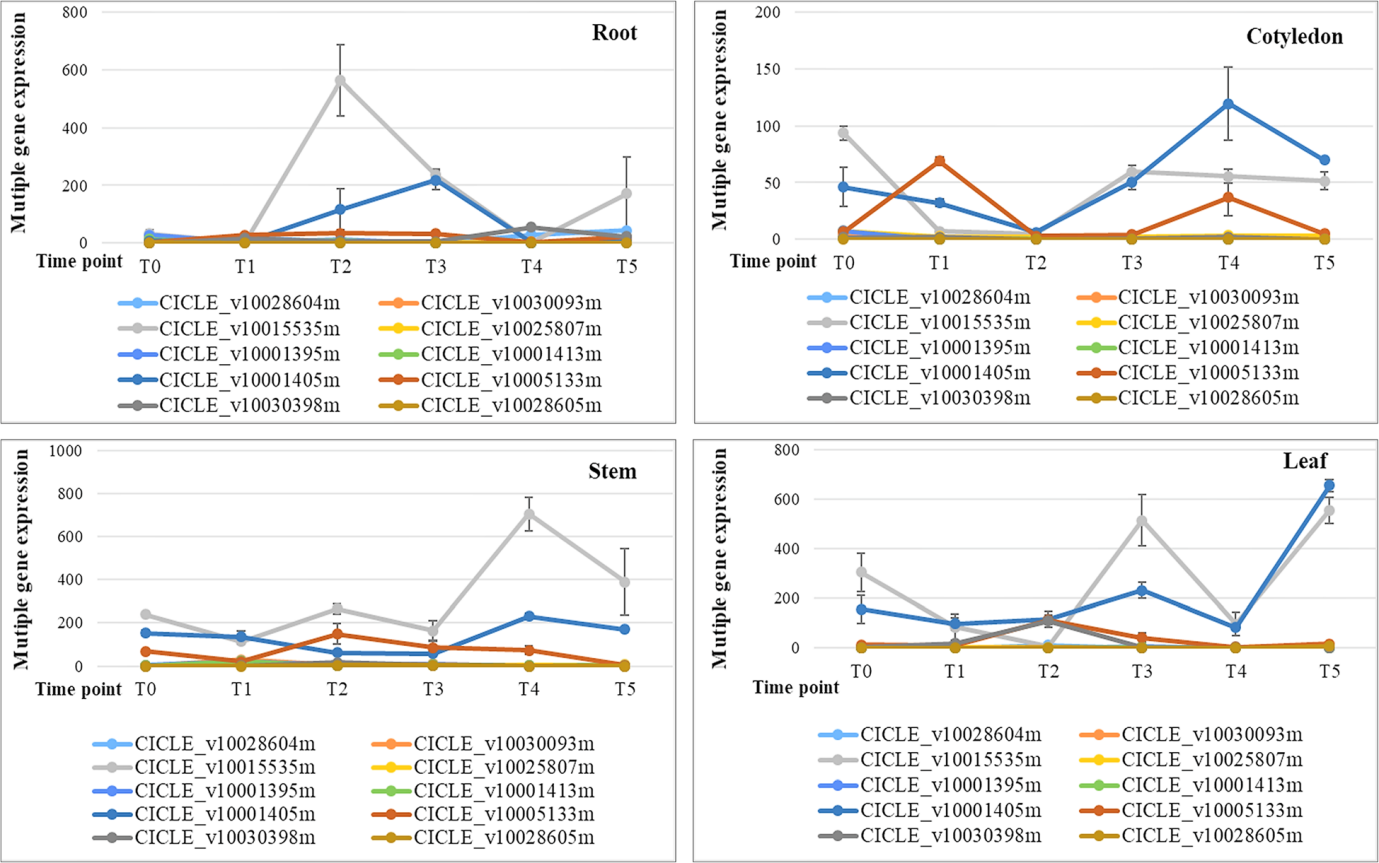
Differential expression patterns of the 10 *CHS* candidates in young seedlings with or without the MeJA treatment The ordinate Y is the relative expression level based on the 2^^(−△△Ct)^ method. The X-axis shows the time points for the MeJA treatment. T0 is the control with distilled water. T1 (12 h) is 12 h after MeJA treatment (same convention for T2 (24 h), T3 (36 h), T4 (48 h), and T5 (60 h))

The expression of the *CHS* genes was tissue specific (Fig. 2). These three genes did not express in the root in the absence of the MeJA treatment. *CitCHS1* was not detected in the cotyledon and leaf before the MeJA treatment. However, all three *CHS* genes were expressed in the stem. MeJA induced the expression of all three genes in the root. The results demonstrated that the three *CHS* genes responded differently to the MeJA treatment. Overall, *CitCHS1* showed the greatest response to the MeJA treatment among the three genes. The expression pattern of the three genes in response to MeJA is tissue specific. In the root, MeJA enhanced the expression of all three genes, particularly *CitCHS2* and *CitCHS3* after the first two MeJA application. *CitCHS2* and *CitCHS3* present similar expression profiles in the stem, cotyledon and leaf. MeJA suppressed the expression of these two *CHS* genes at the early stage of treatment but enhanced the expression at the late stage. *CitCHS1* showed similar expression pattern as the other two genes in the stem, although its expression was enhanced in the cotyledon and leaf.

### MeJA treatment induced the production of flavonoids

**Fig. 3:**
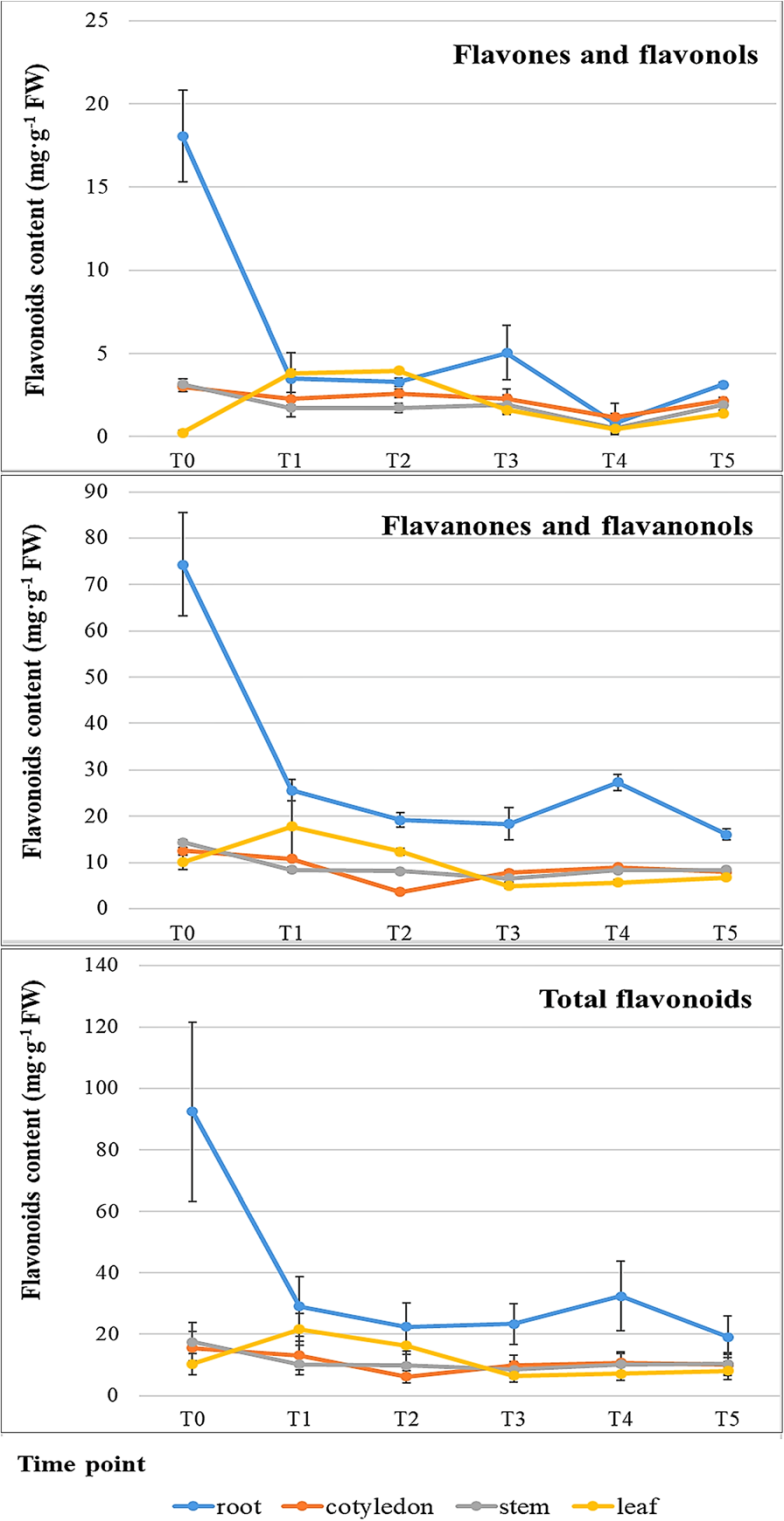
Flavonoid contents in young seedlings with or without the MeJA treatment The total flavonoids are calculated by the sum of the contents of the four dominant components. T0 is the control with distilled water treatment. T1 (12 h) is 12 h after MeJA treatment (same convention for T2 (24 h), T3 (36 h), T4 (48 h), and T5 (60 h))

To investigate the distribution of total flavonoids in the four tissues, the contents of four main types of flavonoids, i.e., flavones, flavonols, flavanones and flavanonols, were analysed in the seedlings. In the roots, the highest contents of each flavonoid component were detected at time T0 before the MeJA treatment. A sharp decrease in flavonoid contents was observed after the MeJA application, and the content reached a very low level at 12 h after spraying (T1) (Fig. 3). The contents of flavonoids did not significantly change from T1 (12 h after treatment) to T5 (60 h after treatment), only showed slight fluctuations; however, the lowest content of each flavonoid appeared at T0 in the leaves. In leaves, MeJA induced the production of flavonoids in the first 12 h after treatment, and this effect gradually declined through T2 until T3. In the cotyledons and stems, the contents of flavonoids had a similar variation tendency as that in the roots, but the level of variation was much less significant.

### Correlation between *CitCHS* expression and flavonoid production under MeJA treatment

To identify the function of the three *CitCHS* genes, the correlation between gene expression and flavonoid accumulation was studied (Table 1). Among the three *CHS* genes, the expression of *CitCHS1* was not positively correlated with the flavonoid content, including the four primary compounds, in most tissues except the roots, which showed a correlation coefficient of 0.77 between the expression level and total flavonoids. The expression level of the other two genes, *CitCHS2* and *CitCHS3*, was positively correlated with individual flavonoid accumulation in the root, cotyledon and stem, but not the leaf. However, the expression level of both *CitCHS2* and *CitCHS3* was found to be correlated with the accumulation of total flavonoids in the leaf. The highest correlation for *CitCHS2* was found in the stem (*R* = 0.81). *CitCHS3* was found to be positively correlated with flavones and flavonols in the root, with flavanones and flavanonols in the cotyledon and stem. Interestingly, the expression of *CitCHS3* was more significantly correlated with the content of flavanones and flavanonols and total flavonoids in the root and cotyledon than was that of *CitCHS2*, although the opposite trend was observed in the stem and leaf. However, the overall expression level of *CitCHS* was highly correlated with the total flavonoid accumulation in the root, cotyledon, stem and leaf. Moreover, the three *CitCHS* genes were co-expressed in the root with coefficients of 0.67, 0.64 and 0.60, respectively. In the other three tissues, co-expression was only found between *CitCHS2* and *CitCHS3*.

**Table 1:** Pearson’s correlation coefficients between gene expression and flavonoid accumulation in four tissues of young seedlings based on time point T0-T5

		FV + FVL	FN + FNL	TF	*CHS1*	*CHS2*	*CHS3*
Root	*CHS1*	−0.18 ^NS^	−0.5 ^NS^	0.77^***^			
	*CHS2*	0.39^***^	0.19 ^NS^	0.48^***^	0.67^***^		
	*CHS3*	0.52^***^	0.26 ^NS^	0.67^***^	0.64^***^	0.6^***^	
	*CHS*s	0.45^***^	0.2 ^NS^	0.59^***^	0.75^***^	0.96^***^	0.79^***^
Cotyledon	*CHS1*	−0.54 ^NS^	0.11 ^NS^	0.05 ^NS^			
	*CHS2*	−0.01 ^NS^	0.49^***^	0.52^***^	−0.37 ^NS^		
	*CHS3*	−0.02 ^NS^	0.62^***^	0.65^***^	0.03 ^NS^	0.5^***^	
	*CHS*s	−0.31 ^NS^	0.58^***^	0.58^***^	0.38^***^	0.59^***^	0.85^***^
Stem	*CHS1*	0.46^***^	0.29 ^NS^	0.34^***^			
	*CHS2*	0.45^***^	0.45^***^	0.8^***^	0.01 ^NS^		
	*CHS3*	−0.09 ^NS^	0.44^***^	0.34^***^	−0.53 ^NS^	0.76^***^	
	*CHS*s	0.42^***^	0.86^***^	0.79^***^	0.07 ^NS^	0.99^***^	0.75^***^
Leaf	*CHS1*	0.01 ^NS^	−0.85 ^NS^	−0.77 ^NS^			
	*CHS2*	−0.64 ^NS^	0.01 ^NS^	0.59^***^	−0.31 ^NS^		
	*CHS3*	−0.07 ^NS^	0.05 ^NS^	0.39^***^	−0.16 ^NS^	0.78^***^	
	*CHS*s	−0.39 ^NS^	−0.05 ^NS^	0.48^***^	−0.16 ^NS^	0.94^***^	0.94^***^

Note: FV + FVL, Flavones and flavonols; FN + FNL, Flavanones and flavanonols; TF, Total flavonoids; *CHS*s, total *CHS*; *CHS1*, *CitCHS1*; *CHS2*, *CitCHS2*; and *CHS3*, *CitCHS3*

NS=Not significantly different at *P* < 0.05; *** = Significant at *P* < 0.001

### Constitution of the *CitCHS* genes

**Fig. 4:**
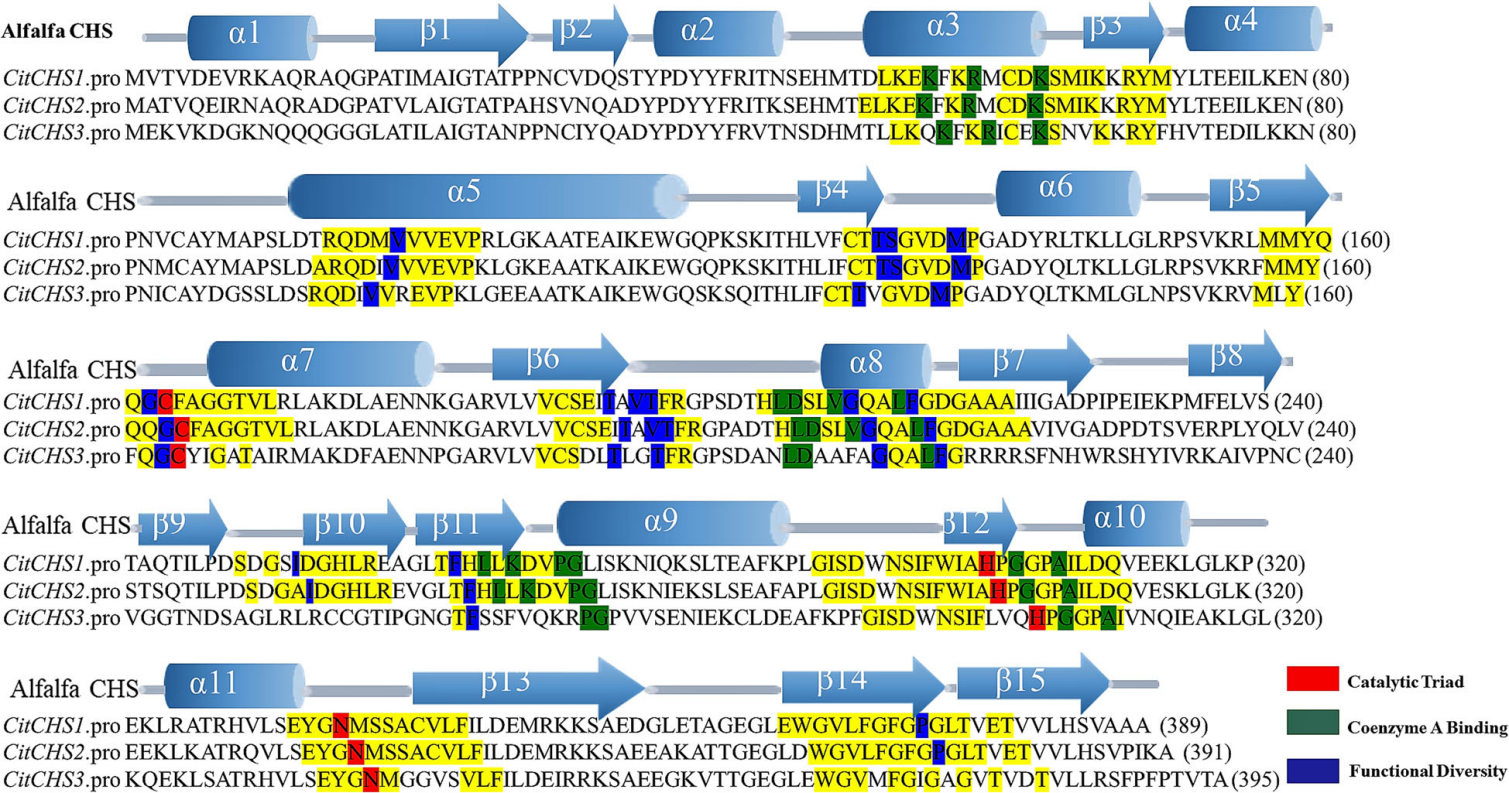
Structure and activity sites of the three CHS genes obtained from citrus This figure was drawn according to Austin and Noel [37]. Key sections are highlighted in yellow. The CHS catalytic triad, residues bound to CoA, and other residues important for functional diversity are highlighted in red, green, and blue, respectively. For clarity, only identical residues in the equivalent positions of the aligned sequences are highlighted

*CitCHS2* (Accession No. KP720583-KP720592) was cloned from ten different citrus species in our previous work [36]. The cDNAs of *CitCHS1* (Accession No. MF784513) and *CitCHS3* (Accession No. MF776052) were amplified from grapefruit (*Citrus paradisi* Macf. cv. Duncan) and ‘Sunred’ (a red-fleshed hybrid of *C. clementina* Oroval × *C. sinensis* Moro blood orange), respectively, in this study. The lengths of the CDS of *CitCHS1*, *CitCHS2* and *CitCHS3* were 1170 bp, 1176 bp and 1194 bp, respectively. The DNA sequence of the three *CHS* genes consisted of one intron and two exons, and the first exon was much smaller than the second. Moreover, the length of the first exon for each of the three *CHS* genes was the same at 180 nucleotides, representing 60 amino acids. The DNA sequence of the three *CHS* genes showed variation in the second exon. Based on the constructed structure of CHS in alfalfa [37], the sequence of amino acids showed that the three CHSs obtained from citrus plants contained almost all the main features of the CHS model structure (Fig. 4). This analysis indicated that the three citrus *CHS* genes are the active *CHS* genes.

### Functional validation of *CitCHS* genes with transgenic plants

**Fig. 5:**
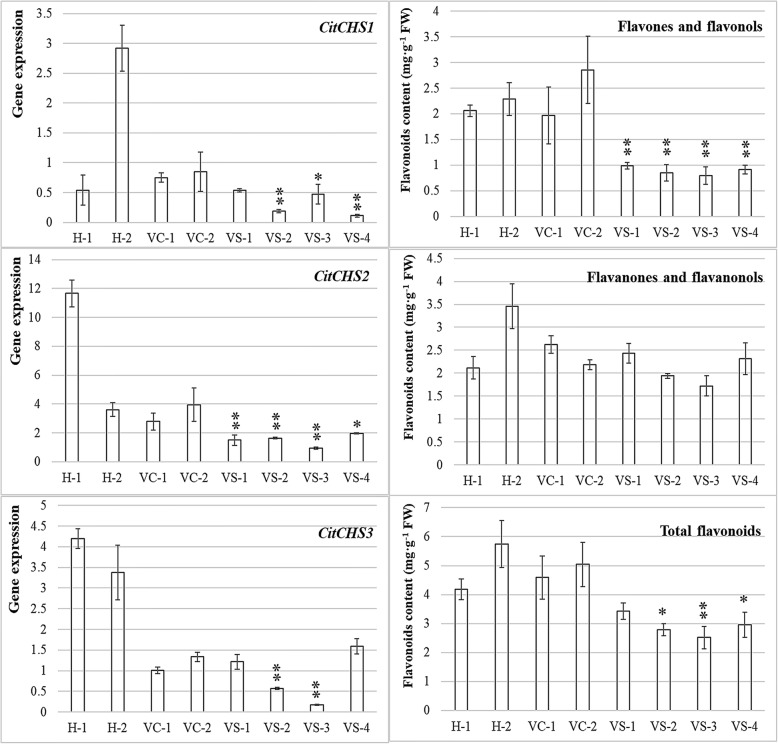
Flavonoid contents and 3 *CHS* expression level in silenced plants ** H-1 and H-2 are healthy control plants, wild type. VC-1 and VC-2 are empty vector control plants. VS-1, VS-2, VS-3 and VS-4 are positive silenced plants. *CitCHS1*, CICLE_v10005133m; *CitCHS2*, CICLE_v10015535m; *CitCHS3*, CICLE_v1001405m. Significant differences with the control (all controls) are indicated: * < 0.05; < 0.01

Virus-induced gene silencing (VIGS) was conducted to validate the function of the three *CitCHS* genes. Four positive plants were selected from the transgenic plants to analyse the correlation between gene expression and flavonoid production. The non-transgenic plants and plants transformed with the empty vector were used as controls. The three *CitCHS* genes showed reduced expression in the plants transformed with an empty vector, at 53.83%, 54.71% and 69.18% of reductions compared with those of the non-transgenic control. However, the reduced gene expression in the empty vector transgenic plants did not result in a significant decrease in the flavonoid content, with only a 3% reduction in total flavonoids. Large differences in the expression of the three *CitCHS* genes were observed in the non-transgenic control plants. *CitCHS2* showed the highest levels of transcripts, whereas *CitCHS1* presented a low level. In all four transgenic plants, three *CitCHS* genes were not completely silenced, although the level of expression was significantly suppressed (Fig. 5). The average levels of suppression of the three *CHS* in transgenic plants were 81.03%, 79.67% and 76.60%. Large variations in the level of suppression were observed among the transgenic plants, although the average level of suppression among the three genes was only slightly different from that of the non-transgenic control, which suggested that VIGS has an equal effect on the three *CitCHS* genes.

The silenced plants produced significantly fewer flavonoids than did the controls. The level of reduction among the four types of flavonoids was different (Fig. 5). Suppressing the expression of the *CitCHS* genes in the silenced plants resulted in a significant reduction of flavone and flavonol production but had less effect on the production of flavonones and flavanonols. However, the total flavonoid production decreased by 41.11% compared with that in the non-transgenic control. The results indicated the importance of *CitCHS* genes for the production of flavonoids.

The contribution of the three *CitCHS* genes towards the production of flavonoids is not similar. The reduced *CHS* gene expression in the empty vector control transgenic plants did not have a lower production of flavonoids. Excluding the empty vector control from analysis, strong correlations of 0.90, 0.43 and 0.80 were observed between the level of gene expression and the total flavonoid content for the three *CitCHS* genes.

**Fig. 6:**
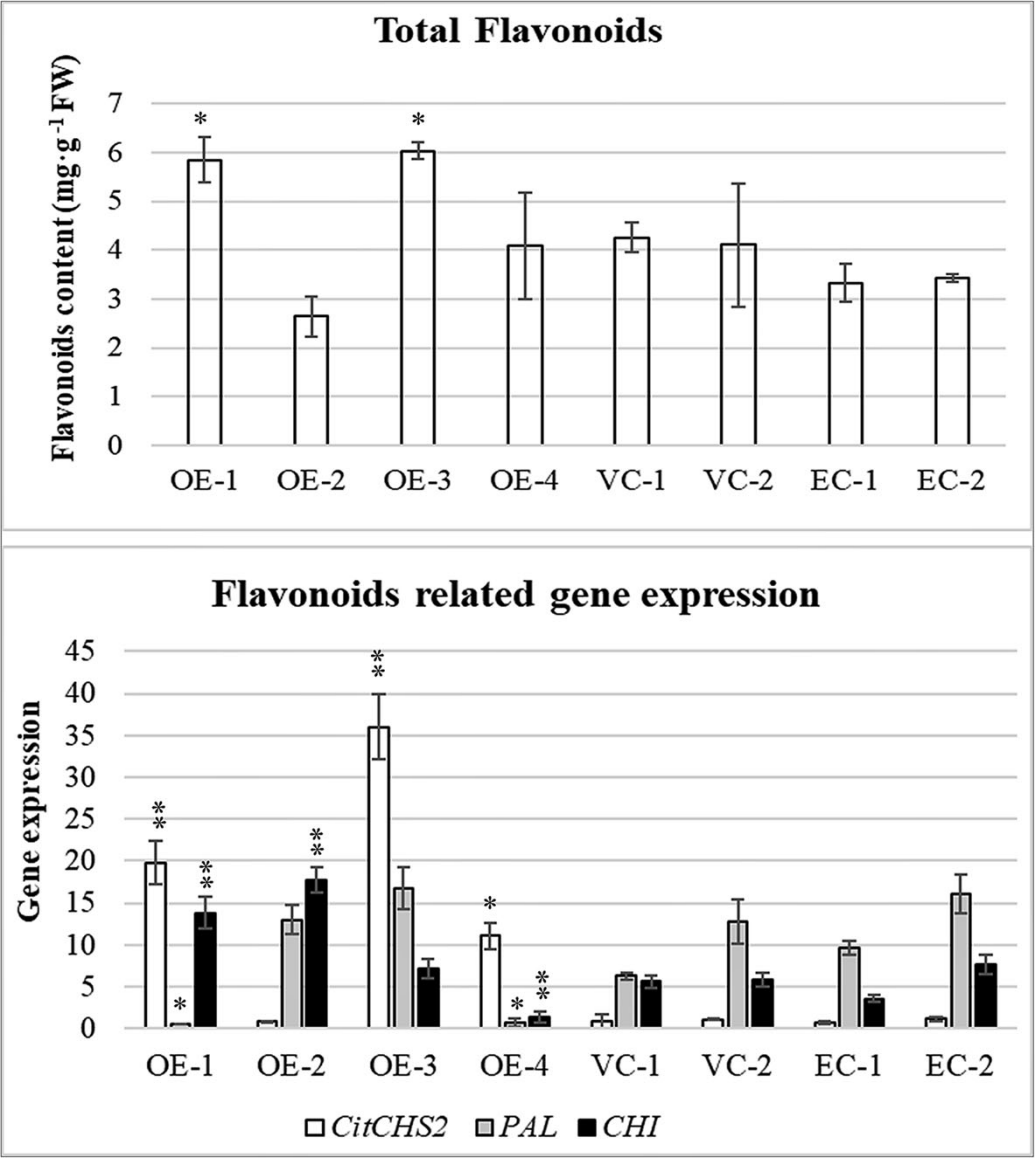
Flavonoid content and expression level of flavonoid production-related genes in over-expression plants ** VC-1 and VC-2 are vector-only control plants. EC-1 and EC-2 are healthy control plants, wild type. OE-1, OE-2, OE-3 and OE-4 are positive over-expression plants. Significant differences with the control (all controls) are indicated: * < 0.05; < 0.01

To identify the contribution of *CHS* genes to the accumulation of flavonoids, four positive overexpression transgenic citrus plants were analysed for both gene expression and flavonoid accumulation. Among the four positive plants, only three (OE-1, OE-3 and OE-4) showed up-regulated *CHS* expression (Fig. 6). An apparent increase in the production of flavonoids was observed in OE plants. The OE-2 plant showed the lowest level of *CHS* expression and flavonoid content among the four OE plants. The *CHS* gene overexpression results also indicated that the *CHS* genes contributed significantly to the production of flavonoids.

## Discussion

The function of the *CHS* gene in controlling flavonoid biosynthesis has been well-documented in many plant species [22–24]. The *CHS* superfamily has also been reported in many plants such as soybean [32], turnip [27] and mulberry [29]. Thus far, studies have not discussed the phenomenon of the *CHS* superfamily or functional members of this family in citrus plants. In this study, the phylogenetic analysis of 77 *CHS* or *CHS*-related genes from the citrus genome revealed that the citrus *CHS* family may include many members. The expression of three *CHS* members from 10 candidates were identified in different tissues of young seedlings, suggesting that they are active in citrus. One of them appeared as a novel *CHS* gene and was termed as *CitCHS3*. Though, the level of transcripts was detected in another gene, CICLE_v10030398m at T4 in root and at T2 in leaf. We did not carry on the further work to characterize the function of this gene in this study because it only temporarily expressed under the MeJA treatment. Plant hormone, like JA/MeJA (Jasmonate/Methyl jasmonate) can modulate CHS gene expression [38]. It was reported that both ABA and JA are vital signalling molecules in plants as they can induce stress-resistance and participate in the formation of systemic resistance, through the wound signal transduction pathway [39]. Certain chemical elicitors, such as ABA, SA, JA can mimic environmental stress [40], induce the expression of *CHS* and enhance the activity of *CHS* in many plants [41–43]. Cross-talk between these chemical signaling pathways is very common in plant responses to abiotic and biotic factors. In the present study, the ‘Sunred’ blood orange hybrid seedlings were treated with MeJA to identify the activity of *CHS* genes. Of 10 selected *CHS* and *CHS*-like genes, the three *CHS* genes including *CHS3* greatly responded to the MeJA treatment. This result confirmed that MeJA could enhance the expression of *CHS* genes. The correlation analysis showed that there is a tight relationship between flavonoid accumulation and *CHS* expression in the MeJA-treated plants. However, the levels of expression of the three *CHS* genes as well as their correlation with the accumulation of flavonoids were different. The expression of all three *CHS* genes demonstrated significant correlations with the accumulation of total flavonoids, indicating the importance of *CHS* for controlling the biosynthesis of flavonoids in citrus.

In citrus, the *CHS* gene appeared as a large gene family, as reported in other plants. Two *CHS* genes (*CitCHS1* and *CitCHS2*) were reported in previous studies [35]. Three copies of the *CitCHS2* gene were identified in *Citrus. sinensis* (L.) Osbeck cv. Ruby. They were located on three different chromosomes [44]. Nine *CHS* genes were studied in ‘Rio Red’ grapefruit (*C. paradisi*.) [24]. These *CHS* genes shared 86–87% and 97–99% similarities with *CitCHS1* and *CitCHS2,* respectively. Presumably, these genes should be the copies of different *CitCHS* members. Only a slight difference was observed in the amino acid sequences among most *CHS* genes in citrus, although variations in their activity in the control of flavonoid biosynthesis were observed. Therefore, we attempted to study the functional characteristics of *CHS* members in this study.

Comparison of genomic DNA sequence, the cDNA structure of the *CHS* gene had an intron and two exons with the same length of 60 amino acids in the first exon and shared over 90% sequence similarity, whereas the second exons were much less conserved, which indicated that the first exon is important for the activity of chalcone synthase because it is part of the basic structure of *CHS*. The novel *CHS* gene identified in this study (i.e., *CitCHS3*) shared high identity with the reported *CHS* and demonstrated a close correlation with the production of flavonoids. This novel *CHS* should represent a new member of the *CitCHS* family, suggesting that the *CHS* gene family in citrus contains at least three functional members and each member may have multiple copies.

Among these three functional members, the expression of *CitCHS1* was not induced by embryogenesis in citrus [35]. Similarly, the present study showed that the expression level of *CitCHS1* was low and maintained a relatively steady level in the four studied tissues compared with the other two *CitCHS* genes. Thus, *CitCHS1* is likely a tissue-specific gene or is not sensitive to MeJA treatment. *CitCHS2* is a well-recognized gene. In this study, the *CitCHS3* gene demonstrated a high level of expression. The correlation analysis also showed that the *CitCHS3* gene was co-expressed closely with *CitCHS2* (*r* > 0.83) and had a high correlation (*r* = 0.6) with the accumulation of total flavonoids in silenced plants (Table 2).

**Table 2:** Pearson’s correlation coefficients between gene expression and flavonoid accumulation in silenced plants

Coefficient analysis	*CitCHS1*		*CitCHS2*		*CitCHS3*		*Total CHS*s	
FV + FVL	0.55	***	0.51	***	0.52	***	0.60	***
FN + FNL	0.86	***	0.02	NS	0.49	***	0.31	***
TF	0.79	***	0.37	***	0.58	***	0.57	***
*CitCHS1*			0.08	NS	0.50	***	0.38	***
*CitCHS2*					0.83	***	0.95	***
*CitCHS3*							0.95	***

Note: FV + FVL, Flavones and flavonols; FN + FNL, Flavanones and flavanonols; TF, Total flavonoids. NS=Not significantly different at *P* < 0.05; *** = Significant at *P* < 0.001

Although the reduction of the *CHS* expression were found in the empty vector control plants with 3% of flavonoids reduction. The reduction of both the *CitCHS* gene expression and the flavonoids contents are significantly correlated in the silenced plants, in comparison with both non-transgenic control and empty vector control plants.

*CHS* is located at an important regulatory point upstream of the flavonoid biosynthetic pathway. It can channel the flux of the phenylpropanoid pathway towards flavonoid biosynthesis [43]. Thus, up- or down-regulation of *CHS* gene expression may strongly affect the production of flavonoids. In previous study, *PAL* (Phenylalanine ammonia-lyase) shared a similar expression pattern with *CHI* [45], though no consistent rules were found regarding *PAL* expression or its influence on *CHS* in pears [46]. We also tried to illuminate the correlation among *PAL*, *CHS* and *CHI*. Our result showed that the overexpression of *CHS* maybe positively affect the expression of *CHI* gene, which is located downstream of *CHS*, but no obvious influence can be found to *PAL* gene, which is located upstream of *CHS* in the flavonoid biosynthesis pathway.

## Conclusions

A novel *CHS* gene named *CitCHS3* (Accession No. MF776052) was identified in citrus plants. *CHS* is a superfamily in the citrus genome with at least three functional genes that can regulate the biosynthesis of flavonoids. Three *CitCHS* genes have unique spatial and temporal expression properties and contribute differently to the production of flavonoids.

## Materials and methods

### Plant materials and methyl jasmonate treatment

Seeds were collected from the mature fruits of ‘Sunred’ blood orange hybrid (*C. clementina* Oroval × *C. sinensis* ‘Moro’) in the field of CREC, UF, on Nov. 2016. The seeds were germinated in soil after the removal of both outer and inner seed coat and grown in a greenhouse under a natural light cycle. Four-week-old seedlings were used in this study. JA/MeJA can modulate *CHS* gene expression [38]. MeJA (Sigma Company, USA) was prepared at a concentration of 200 μM according to the method of Shi [47]. The seedlings were sprayed with MeJA every 12 h immediately after sampling. The control was sprayed with distilled water. The leaf, stem, cotyledon and root samples were collected every 12 h. T0 is the control without any treatment (only water). T1 (12 h) is 12 h after treatment (same convention for T2 (24 h), T3 (36 h), T4 (48 h), and T5 (60 h)). Samples from 10 to 15 seedlings were mixed together, with three replications performed for each time point. The samples were immediately rinsed in distilled water, placed into liquid nitrogen for freezing, and then stored at − 80 °C for further use. The samples were ground into a fine powder in liquid nitrogen for both RNA extraction (Agilent Plant RNA Isolation Kit (Agilent, USA)) and flavonoids detection.

### Total RNA isolation and cDNA synthesis

Total RNA was extracted according to the protocol of the Agilent Plant RNA Isolation Kit (Agilent, USA). The integrity and concentration of RNA were determined via 2.0% agarose gel electrophoresis and a NanoDrop 2000 spectrophotometer (Thermo, Waltham, MA, U.S.A.), respectively. One microgram of DNA-free RNA was initiated using a mixed primer (oligo (dT): random primer = 1.7:0.3, V: V, concentration: 10 μM) for first-strand cDNA synthesis with an Affinity Script QPCR cDNA Synthesis Kit (Agilent, USA) following the manufacturer’s instructions. The product was diluted in a 4-fold volume of sterile deionized water and stored at − 20 °C.

### Expression analysis

The relative expression of ten candidate *CHS* genes selected from the phylogenetic analysis was evaluated via qRT-PCR with SYBR Green QPCR Master Mix (Agilent, USA). The qPCR analysis was performed with a CFX96TM Real-Time System (Bio-Rad, USA) in a total volume of 20 μL containing 10 μL of 2× SYBR Green QPCR Master Mix (Agilent, USA), 0.1 μM specific primers (each), and 10 ng of cDNA template. The RNA used in this experiment were extracted through Agilent Plant RNA Isolation Kit (Agilent, USA). The reaction mixtures were heated to 95 °C for 3 min, followed by 40 cycles at 95 °C for 15 s, 60 °C for 15 s, and 72 °C for 20 s. The differences in gene expression were calculated using the 2^^(−△△Ct)^ analysis method. The level of transcription was determined by relative quantification using the citrus GAPDH gene as the reference gene [48]. Three different RNA (of three separated biological replicates) isolations and cDNA syntheses were used as replicates for the qRT-PCR.

### Isolation of *CHS* genes

Genomic DNA and total RNA were extracted from the young leaves of citrus plants using the CTAB method. RNA was extracted according to the protocol of the Agilent Plant RNA Isolation Kit (Agilent, USA). Gene specific primers were designed using NCBI online primer-design software (https://www.ncbi.nlm.nih.gov/tools/primer-blast/). The RT-PCR reactions were conducted through a program of 95 °C for 5 min, 58 °C for 25 s, and 72 °C for 1 min, 35 cycles, at last 5 min time for extension. All the PCR products were purified through the QIAquick Gel Extraction Kit (QIAGEN, US) and cloned into p-EASY vectors (Transgene, China). The positive clones were sent for sequencing in Eton Bioscience Company (US). The acquired sequences were submitted to Genbank.

### Flavonoid detection

The method of detecting flavones and flavonols was modified from the aluminium chloride colourimetric method reported by Woisky and Sllatino [49] and Chang [50]. The criterion solutions were generated via step by step dilution with standard quercetin and chromatography-grade methanol in consecutive concentrations of 1000 μg/mL, 500 μg/mL, 250 μg/mL, 100 μg/mL, 50 μg/mL and 25 μg/mL. The absorbance was measured at 415 nm with a Benchmark Plus microplate spectrophotometer (Bio-Rad, USA). The standard curve is in Additional file 1: Table S3-A. One gram of powdered sample was extracted twice with methanol. The first extraction was with 10 mL methanol, and the second was with 5 mL methanol. Each extraction was incubated at 50 °C and subjected to shaking at 200 rpm for 30 min. The residual was removed by centrifugation at 10,000 r/min for 10 min. After a final centrifugation at 10,000 r/min for 10 min, the 0.5 mL of methanol extract was reacted with aluminium chloride before measuring the absorbance at 415 nm with the spectrophotometer.

The protocol for detecting flavanones and flavanonols was slightly modified from the method described by Chang [30]. Naringenin was used as a standard chemical to generate criterion solutions at concentrations of 50, 100, 200, 500, 1000, 3000, 4000 and 5000 μg/mL with methanol. The absorbance was measured at 495 nm. The standard curve is in Additional file 1: Table S3-B. One gram of the powdered sample was extracted twice with methanol as described above and reacted with 2,4-dinitrophenylhydrazine, and the flavonoid content was determined by measuring the absorbance at 495 nm.

### Overexpression of *CitCHS2*

**Fig. 7:**
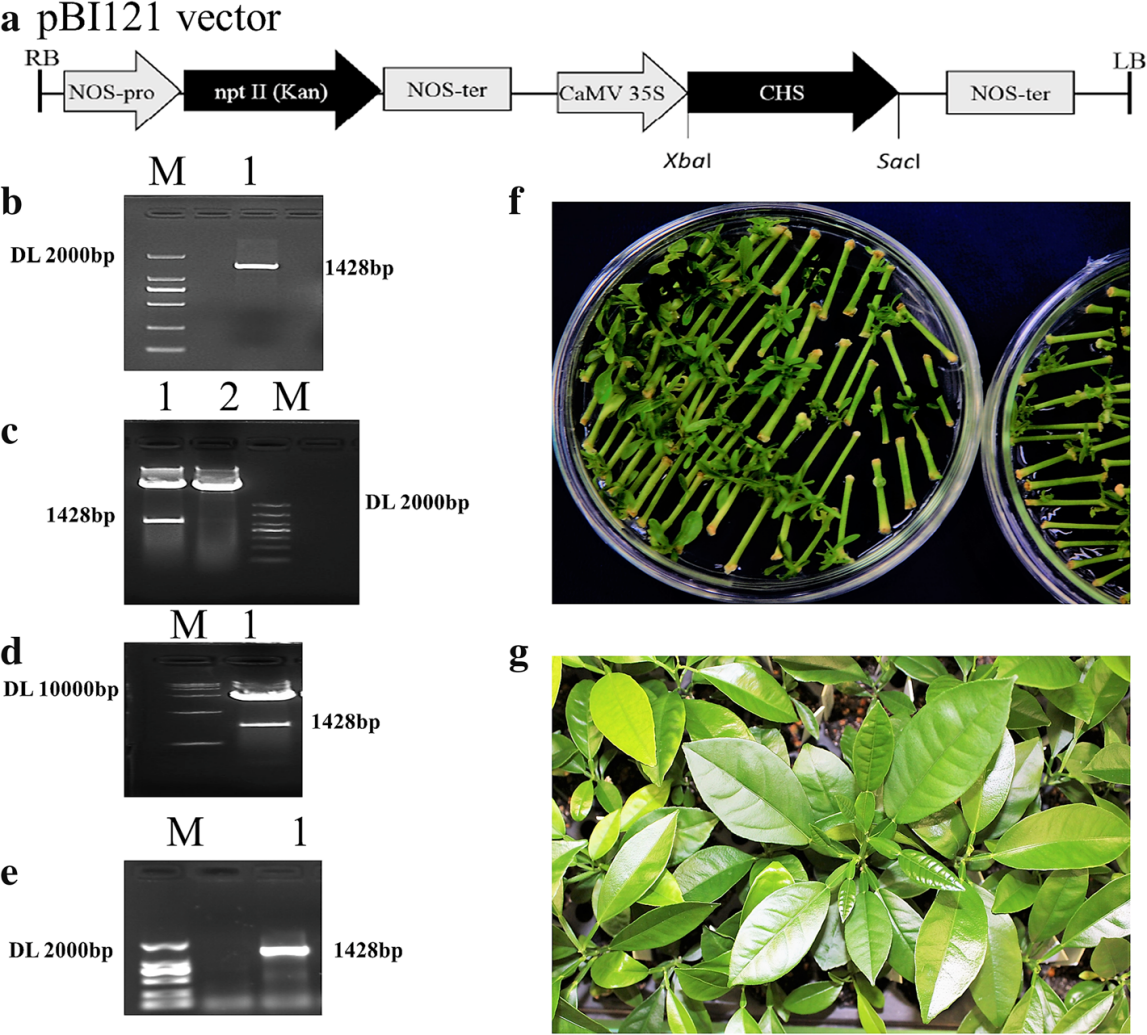
Overexpression of the *CitCHS2* gene in citrus plants **a**:1428 bp fragment containing the *CitCHS2* gene ORF was constructed in the pBI121 vector. **b**: 1428 bp fragment was cloned via RT-PCR. M, marker; 1, 1428 bp fragment contain the ORF of *CitCHS2*. **c**: Pattern of double digestion by *Xba*I and *Sac*I of the p-EASY vector containing the 1428 bp fragment and the pBI121 vector. M, marker; 1, the p-EASY vector containing the 1428 bp fragment; 2, the pBI121 vector. **d**:Validation of the constructed pBI121 and *CitCHS2* ORF vector via double digestion by *Xba*I and *Sac*I. M, marker; 1, the constructed pBI121 vector. **e**: Colony PCR of the positive *Agrobacterium tumefaciens* strain EHA 105 that contains the constructed pBI121 vector. M, marker; 1, the positive colony PCR product. **f**: Callus generated from the epicotyl of *C. sinensis* after the infection of the *Agrobacterium tumefaciens*. **g**: Grafted positive plants grown on the rootstock

The complete ORF fragment of the *CitCHS2* gene was amplified from citrus cDNA via PCR using a gene-specific primer with added *Xba*I and *Sac*I sites cloned into a p-EASY vector (Transgene, China) (Fig. 7a and b). After confirmation by sequencing, the constructed vector was digested with the *Xba*I and *Sac*I enzymes. Moreover, the PBI121 (Clontech Laboratories, USA) vector was also digested with *Xba*I and *Sac*I (Fig. 7c). The digested fragments were separated on a 2% agarose gel. The ORF fragment of the *CitCHS2* gene was fused to the PBI121 vector with T4 DNA ligase (Transgene, China). The plasmid was transformed into a disarmed strain of *Agrobacterium tumefaciens*, EH105. An empty vector of PBI121 was also transformed into EH105 as a control. The 4-week-old epicotyl of sweet orange (*C. sinensis*) with the same phenological period was transformed by Agrobacterium infection according to the method described by Horsch et al. [51].

### Virus-induced *CHS* gene silencing

**Fig. 8:**
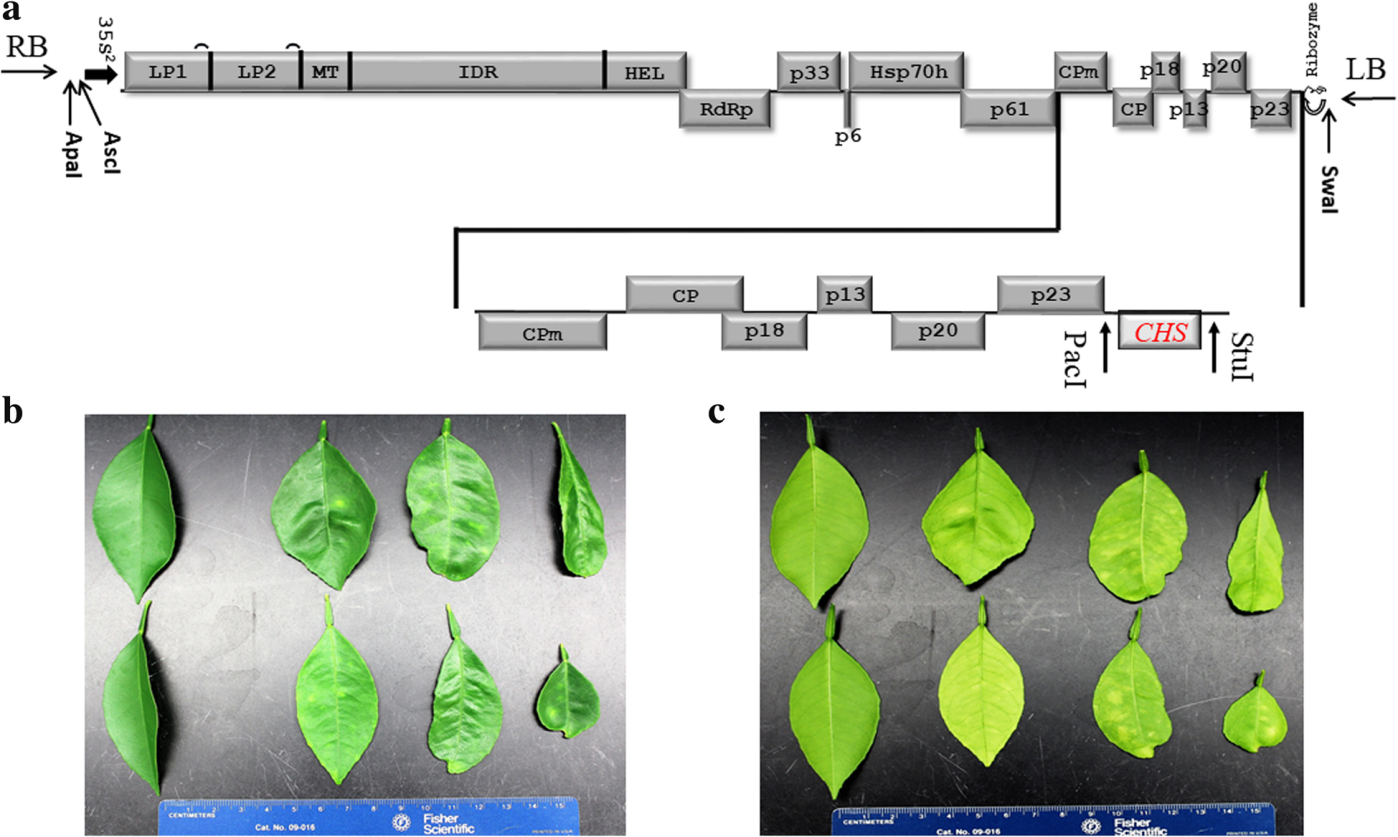
Virus-induced gene silencing **a**: Sketch of the cDNA genome of CTV in the pCAMBIA 1380 vector and position of the *CHS* fragment. **b**: Symptom of the leaf after inoculation, with the first column representing the control. **c**: Symptoms in the leaf after the inoculation, with the first column representing the control

Gene-specific primers were designed from a highly conserved region of the *CitCHS2* gene. A 345-nucleotide sequence was amplified for gene silencing. The fragment was fused to a CTV vector (Fig. 8a). The *Agrobacterium tumefaciens* strain EHA 105 was transformed with the binary plasmid containing CTV, the target gene fragment and silencing suppressors. *Nicotiana benthamiana* plants were used for infection to maximize the virus titre. Then, one-year-old ‘Pineapple’ orange trees with the same phenological period were used for inoculation infection with virions partially purified from the sap derived from agroinfiltrated *N. benthamiana* leaves [52]. The distribution of the CTV vector in the leaves of both the lower and upper parts of the plant was confirmed by ELISA according to the protocol of Garnsey [53]. A double antibody sandwich indirect enzyme-linked-immunosorbent assay (DAS-I-ELISA) [53] was used with purified IgG from rabbit polyclonal antibody CTV-908 (1 μg/ml) for coating, and a broadly reactive CTV Mab172 was used for detection. Total RNA was extracted from the leaves in the upper part of the inoculated citrus plants, and the synthesis of first-strand cDNA was performed as previously described. The integrity of the cDNA and silencing sequence were confirmed via PCR.

### Correlation analysis

The correlation analysis was conducted through Pearson’s correlation analysis. Three separated biological replicates in all time points (including T0) were used for analysis.

## Additional files

Additional file 1: **Table S1.** Identify homology of CHS or CHS-like genes considered in Fig. 1. **Table S2.** Information of the CHS candidate genes and primers used for expression analysis. **Table S3.** Standard curve used in flavonoids detection. (DOCX 127 kb)

## Abbreviations

CDS: Coding Sequence
CHI: Chalcone isomerase
CHS: Chalcone synthase
CoA: Coenzyme A
MeJA: Methyl jasmonate
PAL: Phenylalanine ammonia-lyase
PKS: Polyketide synthase
VIGS: Virus induced gene silencing

## References

[1] Liu Y, Lou Q, Xu W, Xin Y, Bassett C, Wang Y (2011). Characterization of a chalcone synthase (CHS) flower-specific promoter from Lilium orential 'Sorbonne. Plant Cell Rep.

[2] Tan J, Wang M, Tu L, Nie Y, Lin Y, Zhang X (2013). The flavonoid pathway regulates the petal colors of Ccotton flower. PLoS One.

[3] Yazaki K, Sugiyama A, Morita M, Shitan N (2008). Secondary transport as an efficient membrane transport mechanism for plant secondary metabolites. Phytochem Rev.

[4] Goławska S, Sprawka I, Łukasik I, Goławski A (2014). Are naringenin and quercetin useful chemicals in pest-management strategies?. J Pest Sci.

[5] Gabriele M, Frassinetti S, Caltavuturo L, Montero L, Dinelli G, Longo V, Di Gioia D, Pucci L (2017). Citrus bergamia powder: antioxidant, antimicrobial and anti-inflammatory properties. J Funct Foods.

[6] Fini A, Brunetti C, Di Ferdinando M, Ferrini F, Tattini M (2011). Stress-induced flavonoid biosynthesis and the antioxidant machinery of plants. Plant Signal Behav.

[7] Peer WA, Murphy AS (2007). Flavonoids and auxin transport: modulators or regulators?. Trends Plant Sci.

[8] Yao LH, Jiang YM, Shi J, FA TÁS-BÁN, Datta N, Singanusong R, Chen SS (2004). Flavonoids in food and their health benefits. Plant Foods Hum Nutr.

[9] Tarahovsky YS, Kim YA, Yagolnik EA, Muzafarov EN (2014). Flavonoid–membrane interactions: involvement of flavonoid–metal complexes in raft signaling. Biochim Biophys Acta Biomembr.

[10] Chidambara Murthy KN, Kim J, Vikram A, Patil BS (2012). Differential inhibition of human colon cancer cells by structurally similar flavonoids of citrus. Food Chem.

[11] Birt DF, Hendrich S, Wang W (2001). Dietary agents in cancer prevention: flavonoids and isoflavonoids. Pharmacol Ther.

[12] Majo DD, Giammanco M, Guardia ML, Tripoli E, Giammanco S, Finotti E (2005). Flavanones in Citrus fruit: structure–antioxidant activity relationships. Food Res Int.

[13] Ghasemi K, Ghasemi Y, Ebrahimzadeh MA (2009). Antioxidant activity, phenol and flavonoid contents of 13 citrus species peels and tissues. Pak J Pharm Sci.

[14] Calabrò ML, Galtieri V, Cutroneo P, Tommasini S, Ficarra P, Ficarra R (2004). Study of the extraction procedure by experimental design and validation of a LC method for determination of flavonoids in Citrus bergamia juice. J Pharm Biomed Anal.

[15] Tripoli E, Guardia ML, Giammanco S, Majo DD, Giammanco M (2007). Citrus flavonoids: molecular structure, biological activity and nutritional properties: a review. Food Chem.

[16] Guimarães R, Barros L, Barreira JCM, Sousa MJ, Carvalho AM, Ferreira ICFR (2010). Targeting excessive free radicals with peels and juices of citrus fruits: grapefruit, lemon, lime and orange. Food Chem Toxicol.

[17] Singanusong R, Nipornram S, Tochampa W, Rattanatraiwong P (2015). Low power ultrasound-assisted extraction of phenolic compounds from mandarin (Citrus reticulata Blanco cv. Sainampueng) and lime (Citrus aurantifolia) peels and the antioxidant. Food Anal Methods.

[18] Barreca D, Bisignano C, Ginestra G, Bisignano G, Bellocco E, Leuzzi U, Gattuso G (2013). Polymethoxylated, C- and O-glycosyl flavonoids in tangelo (Citrus reticulata × Citrus paradisi) juice and their influence on antioxidant properties. Food Chem.

[19] Tanaka Y, Brugliera F, Chandler S (2009). Recent progress of flower colour modification by biotechnology. Int J Mol Sci.

[20] Buer CS, Imin N, Djordjevic MA (2010). Flavonoids: new roles for old molecules. J Integr Plant Biol.

[21] Kreuzaler F, Hahlbrock K (1972). Enzymatic synthesis of aromatic compounds in higher plants: formation of naringenin (5, 7, 4′-trihydroxyflavanone) from p-coumaroyl coenzyme a and malonyl coenzyme a. FEBS Lett.

[22] Lewinsohn E, Britsch L, Mazur Y, Gressel J (1989). Flavanone glycoside biosynthesis in citrus chalcone synthase, UDP-glucose: flavanone-7-O-glucosyl-transferase and-rhamnosyl-transferase activities in cell-free extracts. Plant Physiol.

[23] Winkel-Shirley B (2001). Flavonoid biosynthesis. A colorful model for genetics, biochemistry, cell biology, and biotechnology. Plant Physiol.

[24] Chaudhary PR, Bang H, Jayaprakasha GK, Patil BS (2016). Variation in key flavonoid biosynthetic enzymes and phytochemicals in 'Rio Red' grapefruit (Citrus paradisi Macf.) during fruit development. J Agric Food Chem.

[25] Martin CR (1993). Structure, function, and regulation of the chalcone synthase. Int Rev Cytol.

[26] Sanjari S, Shobbar ZS, Ebrahimi M, Hasanloo T, Sadat-Noori S-A, Tirnaz S (2015). Chalcone synthase genes from milk thistle (Silybum marianum): isolation and expression analysis. J Genet.

[27] Zhou B, Wang Y, Zhan Y, Li Y, Kawabata S (2013). Chalcone synthase family genes have redundant roles in anthocyanin biosynthesis and in response to blue/UV-A light in turnip (Brassica rapa; Brassicaceae). Am J Bot.

[28] Yahyaa M, Ali S, Davidovich-Rikanati R, Ibdah M, Shachtier A, Eyal Y, Lewinsohn E, Ibdah M (2017). Characterization of three chalcone synthase-like genes from apple (Malus × domestica Borkh.). Phytochemistry.

[29] Wang C, Zhi S, Liu C, Xu F, Zhao A, Wang X, Tang X, Li Z, Huang P, Yu M (2017). Isolation and characterization of a novel chalcone synthase gene family from mulberry. Plant Physiol Biochem.

[30] Tsai CJ, Ei Kayal W, Harding SA (2006). Populus, the new model system for investigating phenyl-propanoid complexity. Int J Appl Sci Eng.

[31] Tuteja JH, Clough SJ, Chan W-C, Vodkin LO (2004). Tissue-specific gene silencing mediated by a naturally occurring chalcone synthase gene cluster in Glycine max. Plant Cell.

[32] Yi JX, Derynck MR, Chen L, Dhaubhadel S (2010). Differential expression of CHS7 and CHS8 genes in soybean. Planta.

[33] Farzad M, Soria-Hernanz DF, Altura M, Hamilton MB, Weiss MR, Elmendorf HG (2005). Molecular evolution of the chalcone synthase gene family and identification of the expressed copy in flower petal tissue of Viola cornuta. Plant Sci.

[34] Koes RE, Spelt CE, van den Elzen PJM, Mol JNM (1989). Cloning and molecular characterization of the chalcone synthase multigene family of Petunia hybrida. Gene.

[35] Moriguchi T, Kita M, Tomono Y, Endo-Inagaki T, Omura M (1999). One type of chalcone synthase gene expressed during embryogenesis Rregulates the flavonoid accumulation in citrus cell cultures. Plant Cell Physiol.

[36] Wang Z, Shen W, Zhu S, Xue Y, Zhao X (2015). Polymorphism and expression of chalcone synthase gene in citrus related to the flavonoids content. Acta Horticulturae Sinica.

[37] Austin MB, Noel JP (2003). The chalcone synthase superfamily of type III polyketide synthases. Nat Prod Rep.

[38] Creelman RA, Tierney ML, Mullet JE (1992). Jasmonic acid/methyl jasmonate accumulate in wounded soybean hypocotyls and modulate wound gene expression. Proc Natl Acad Sci.

[39] Deng WH, Shen YS, Chen HJ, Li ZY, Jiang XN (2009). Effects of Dendrolimus punctatus feeding and methyl jasmonate (MeJA)-or terpenes fumigation on abscisic acid and jasmonic acid contents in Pinus massoniana seedling needles. Chin J Appl Ecol.

[40] Tuteja N, Sopory SK (2008). Chemical signaling under abiotic stress environment in plants. Plant Signal Behav.

[41] Tamari G, Borochov A, Atzorn R, Weiss D (1995). Methyl jasmonate induces pigmentation and flavonoid gene expression in petunia corollas: a possible role in wound response. Physiol Plant.

[42] Sánchez-Sampedro MA, Fernández-Tárrago J, Corchete P (2005). Yeast extract and methyl jasmonate-induced silymarin production in cell cultures of Silybum marianum (L.) Gaertn. J Biotechnol.

[43] Awasthi P, Mahajan V, Jamwal VL, Kapoor N, Rasool S, Bedi YS, Gandhi SG (2016). Cloning and expression analysis of chalcone synthase gene from Coleus forskohlii. J Genet.

[44] Lu X, Zhou W, Gao F (2009). Cloning, characterization and localization of CHS gene from blood orange, Citrus sinensis (L.) Osbeck cv. Ruby. Mol Biol Rep.

[45] Chaudhary PR, Bang H, Jayaprakasha GK, Patil BS (2017). Effect of ethylene degreening on flavonoid pathway gene expression and phytochemicals in Rio red grapefruit (Citrus paradisi Macf). Phytochem Lett.

[46] Yang YN, Yao GF, Zheng D, Zhang SL, Wang C, Zhang MY, Wu J (2015). Expression differences of anthocyanin biosynthesis genes reveal regulation patterns for red pear coloration. Plant Cell Rep.

[47] Shi J, Wang L, Ma CY, Lv HP, Chen ZM, Lin Z (2014). Aroma changes of black tea prepared from methyl jasmonate treated tea plants. J Zhejiang Univ Sci B.

[48] Mafra V, Kubo KS, Alves-Ferreira M, Ribeiro-Alves M, Stuart RM, Boava LP, Rodrigues CM, Machado MA (2012). Reference genes for accurate transcript normalization in citrus genotypes under different experimental conditions. PLoS One.

[49] Woisky RG, Salatino A (1998). Analysis of propolis: some parameters and procedures for chemical quality control. J Apic Res.

[50] Chang CC, Yang MH, Wen H-M, Chern JC (2002). Estimation of total flavonoid content in propolis by tTwo complementary colorimetric methods. J Food Drug Anal.

[51] Horsch RB, Fry JE, Hoffman NL, Eichholtz D, Rogers SC, Fraley RT (1985). A simple and general method for transferring genes into plants. Science.

[52] Gowda S, Satyanarayana T, Robertson C, Garnsey S, Dawson W (2005). Infection of citrus plants with virions generated in Nicotiana benthamiana plants agroinfiltrated with a binary vector based Citrus tristeza virus. International Organization of Citrus Virologists Conference Proceedings (1957-2010).

[53] Garnsey S: Enzyme-linked immunosorbent assay (ELISA) for citrus pathogens. Graft-transmissible diseases of citrus. Handbook for detection and diagnosis1991:193–216.

