# Supplementary material for: Functional study of CHS gene family members in citrus revealed a novel CHS gene affecting the production of flavonoids
Source: BMC Plant Biol. 2018 Sep 12;18:189. doi: 10.1186/s12870-018-1418-y (PMC6134715; doi:10.1186/s12870-018-1418-y)
Supplement: Supplementary file 1 — Table S1. Identify homology of CHS or CHS-like genes considered in Fig. 1. Table S2. Information of the CHS candidate genes and primers used for expression analysis. Table S3. Standard curve used in flavonoids detection. (DOCX 127 kb) [file 12870_2018_1418_MOESM1_ESM.docx]

**Table S1. Identify homology of *CHS* or *CHS*-like genes considered in Figure 1.**

| **Percent Similarity (%)** | CICLE_v10015535m | CICLE_v10005133m | CICLE_v10025807m | CICLE_v10001405m | CICLE_v10001395m | CICLE_v10030398m | CICLE_v10030093m | CICLE_v10001413m | CICLE_v10028605m | CICLE_v10028604m |
| --- | --- | --- | --- | --- | --- | --- | --- | --- | --- | --- |
| Cs2g14720.1 | 88 | 81.5 | 75 | 84.2 | 82.1 | 85.5 | 83.3 | 79.3 | 82.7 | 82.1 |
| Cs1g03730.1 | 75.3 | 77.9 | 88 | 83 | 80.2 | 75.3 | 76.5 | 80.2 | 81.6 | 75.9 |
| orange1.1g036844m | 85.2 | 76.6 | 75.3 | 84.6 | 78.8 | 87 | 81.5 | 78.2 | 80 | 79.5 |
| orange1.1g016111m | 81 | 79.5 | 82.3 | 83 | 83.5 | 80.8 | 81.2 | 86.1 | 85.1 | 84.4 |
| orange1.1g044410m | 77.2 | 76 | 78.4 | 79.7 | 79.2 | 76.9 | 77.2 | 78.7 | 85.4 | 75.3 |
| orange1.1g016330m | 87.8 | 81.5 | 75 | 84.2 | 82.1 | 85.5 | 83.3 | 79.3 | 82.7 | 82.1 |
| orange1.1g016094m | 83.8 | 79.2 | 82.7 | 87.1 | 83.3 | 83.1 | 84.8 | 85.9 | 88 | 83.8 |
| orange1.1g0016081m | 81.9 | 75.6 | 80.5 | 80.7 | 84 | 82.5 | 81.5 | 84.8 | 81.5 | 85.2 |
| NM_001320057 | 88 | 81.5 | 75 | 84.2 | 82.1 | 85.5 | 83.3 | 79.3 | 82.7 | 82.1 |
| NP_001306986 | 87.8 | 81.5 | 75 | 84.2 | 82.1 | 85.5 | 83.3 | 79.3 | 82.7 | 82.1 |
| CICLE_v10025804m | 76.5 | 76.6 | 88 | 84.9 | 81.2 | 75.3 | 76.5 | 80.2 | 82.9 | 75.9 |
| CICLE_v10003127m | 85.3 | 82.4 | 86.8 | 87 | 88.1 | 85.1 | 86.6 | 88.2 | 87 | 86.8 |
| CICLE_v10015535m |  | 85.2 | 76.5 | 84.2 | 82.1 | 85.7 | 85.7 | 79.5 | 84 | 79.8 |
| CICLE_v10005133m |  |  | 76.6 | 76 | 79 | 78.2 | 80 | 77.8 | 80.5 | 75.3 |
| CICLE_v10025807m |  |  |  | 84.9 | 81.2 | 75.3 | 76.5 | 80.2 | 82.9 | 75.9 |
| CICLE_v10001405m |  |  |  |  | 85.7 | 83.3 | 83.6 | 84.2 | 87.1 | 82.8 |
| CICLE_v10001395m |  |  |  |  |  | 79.5 | 80.7 | 81.7 | 83.3 | 81.5 |
| CICLE_v10030398m |  |  |  |  |  |  | 84.5 | 80.2 | 82.1 | 79 |
| CICLE_v10030093m |  |  |  |  |  |  |  | 82.9 | 84.8 | 81.7 |
| CICLE_v10001413m |  |  |  |  |  |  |  |  | 86.1 | 85.4 |
| CICLE_v10028605m |  |  |  |  |  |  |  |  |  | 82.7 |
| CICLE_v10028604m |  |  |  |  |  |  |  |  |  |  |

The sequence distances of 22 *CHS* or *CHS*-like gene considered in figure 1 was worked out through DNAstar, ClustalW (Slow/Accurate, Gonnet), based on the amino acid sequences.

**Table S2. Information of the *CHS* candidate genes and primers used for expression analysis.**

| Sequence name | Accession number | Transcript name or ID (represent) | Transcript name or ID (all) | qPCR primer | product length / bp |
| --- | --- | --- | --- | --- | --- |
| CitCHS1 | MF784513 | Ciclev10005133m.g | CICLE_v10005133m.g | 5'-AAGTCGAGGAGAAGTTGGGC-3' | 88 |
|  |  |  |  | 5'-CAAGCACTCGACATGTTGCC-3' |  |
| Ch8Cl |  | Ciclev10030093m.g | CICLE_v10030093m.g | 5'-CAAGCGGTTTTCGGTGATGG-3' | 150 |
|  |  |  | Orange1.1g044410m | 5'-TTCTTTCAATTGGCCCGAGA-3' |  |
| Ch8C |  | Ciclev10030398m.g | CICLE_v10030398m.g | 5'-TTCGGATCAAAGGCGAGAGG-3' | 118 |
|  |  |  | Orange1.1g036844m | 5'-TGACGAGCATCCAAAGACGG-3' |  |
| CitCHS2 | KP720583—720592 | Ciclev10015535m.g | CICLE_v10015535m.g | 5'-CGGAGATCACGGCAGTCATCT-3' | 87 |
|  |  |  | Cs2g14720.1 |  |  |
|  |  |  | NM_001320057 |  |  |
|  |  |  | NP_001306986 |  |  |
|  |  |  | Orange1.1g016330m | 5'-CAGCACCATCACCGAACAAA-3' |  |
| Ch1C |  | Ciclev10025807m.g | CICLE_v10025807m.g | 5'-ATTCTGGATTGCTCACCCCG-3' | 77 |
|  |  |  | Cs1g03730.1 |  |  |
|  |  |  | CICLE_v10025804m | 5'-ACTTGTCCTTTCTCAGGCCG-3' |  |
| Ch3C |  | Ciclev10001395m.g | CICLE_v10001395m.g | 5'-TAGTTGATGCGGTGAGTCCG-3' | 139 |
|  |  |  |  | 5'-TGTCGGCTTGCCCTTAGTTT-3' |  |
| Ch8C-1 |  | Ciclev10028604m.g | CICLE_v10028604m.g | 5'-ATGGGAGGGACAGTCCTCAG-3' | 144 |
|  |  |  | Orange1.1g016081m | 5'-TGCCTGCACAACCAAGATGT-3' |  |
| Ch3C-1 |  | Ciclev10001413m.g | CICLE_v10001413m.g | 5'-GAGGCCCTGCTGCAATTCTA-3' | 103 |
|  |  |  |  | 5'-TGCTCGCTTTTGGTAACACTG-3' |  |
| Ch8C-2 |  | Ciclev10028605m.g | CICLE_v10028605m.g | 5'-TGTCCCCAACACCGAAAACA-3' | 132 |
|  |  |  | Orange1.1g016094m | 5'-ATCGGACTCATTGCATCAGC-3' |  |
| CitCHS3 | MF776052 | Ciclev10001405m.g | CICLE_v10001405m.g |  | 127 |
|  |  |  | Orange1.1g016111m | 5'-GGCCTCAACCCATCTGTCAA-3' |  |
|  |  |  | CICLE_v10003127m | 5'-AGCATACGACTAGAACGCGG-3' |  |

The sequences were named according to the annotation in NCBI. ‘Ch’ represents for chromosome, ‘C’ represents for CHS. The gb accession number listed here is submitted by us.

**Table S3. Standard curve used in flavonoids detection.**

Curve A was used in flavones and flavonols detection and curve B was used in flavanones and flavanonols detection. The X coordinate axis represents the concentration of the stantard substance and the Y axial represents

the absorbance value of the extraction solution.
